# Analysis of Neat Biofluids Obtained During Cardiac Surgery Using Nanoparticle Tracking Analysis: Methodological Considerations

**DOI:** 10.3389/fcell.2020.00367

**Published:** 2020-05-25

**Authors:** Andrew I. U. Shearn, Sezin Aday, Soumaya Ben-Aicha, Pauline Carnell-Morris, Agnieszka Siupa, Gianni D. Angelini, Aled Clayton, Chantal Boulanger, Prakash Punjabi, Costanza Emanueli, Giovanni Biglino

**Affiliations:** ^1^Bristol Heart Institute, Bristol Royal Infirmary, University of Bristol, Bristol, United Kingdom; ^2^National Heart and Lung Institute, Imperial College London, London, United Kingdom; ^3^Malvern Instruments Ltd., Malvern, United Kingdom; ^4^School of Medicine, Cardiff University, Cardiff, United Kingdom; ^5^Cardiovascular Research Center, INSERM U970, Hôpital Européen Georges Pompidou, Paris, France

**Keywords:** cardiac surgery, exosome (vesicle), exosomal biomarkers, nanopartcicles, NTA (nanoparticle tracking analysis)

## Abstract

Small extracellular vesicles (sEVs) are those nanovesicles 30–150 nm in size with a role in cell signalling and potential as biomarkers of disease. Nanoparticle tracking analysis (NTA) techniques are commonly used to measure sEV concentration in biofluids. However, this quantification technique can be susceptible to sample handing and machine settings. Moreover, some classes of lipoproteins are of similar sizes and could therefore confound sEV quantification, particularly in blood-derived preparations, such serum and plasma. Here we have provided methodological information on NTA measurements and systematically investigated potential factors that could interfere with the reliability and repeatability of results obtained when looking at neat biofluids (i.e., human serum and pericardial fluid) obtained from patients undergoing cardiac surgery and from healthy controls. Data suggest that variables that can affect vesicle quantification include the level of contamination from lipoproteins, number of sample freeze/thaw cycles, sample filtration, using saline-based diluents, video length and keeping the number of particles per frame within defined limits. Those parameters that are of less concern include focus, the “Maximum Jump” setting and the number of videos recorded. However, if these settings are clearly inappropriate the results obtained will be spurious. Similarly, good experimental practice suggests that multiple videos should be recorded. In conclusion, NTA is a perfectible, but still commonly used system for sEVs analyses. Provided users handle their samples with a highly robust and consistent protocol, and accurately report these aspects, they can obtain data that could potentially translate into new clinical biomarkers for diagnosis and monitoring of cardiovascular disease.

## Background

Small extracellular vesicles (sEVs) are generally in the region of 30–150 nm in size and are released from all cells. They are a heterogeneous group of nanoparticles of different cargo content and function, including exosomes (Giulietti et al., 2018). They have become of great interest due to their emerging role in cell-to-cell communication (Théry et al., 2002; Emanueli et al., 2015), through their ability to transfer their active molecular cargoes to recipient cells. Additionally, they can use surface proteins to activate cell signalling pathways in the cells they come into contact with. However, given their small size, established methods, such as standard flow cytometry, which can, for example, be applied to identify and quantify larger EVs, cannot be used directly to investigate concentrations of small EVs (Emanueli et al., 2015; Coumans et al., 2017). In recent years a technique originally developed for other industrial applications, nanoparticle tracking analysis (NTA), has been adapted for this purpose (Dragovic et al., 2011). NTA has become a staple technique in the toolbox of sEV researchers, with the recognition that certain procedures have to be followed to ensure reliable and repeatable data are produced (Filipe et al., 2010; Gardiner et al., 2013; Maas et al., 2015; Tian et al., 2016; Parsons et al., 2017; Vestad et al., 2017). Parsons et al. (2017) showed that the precision of routine NTA measurements can be significantly improved for different biofluids by increasing video replicates. Other studies proposed the use of silica microspheres for calibration to overcome differences between instruments with different specifications (Gardiner et al., 2013) or the use of instrument-optimised settings to correct significantly different results observed between different instruments with identical software settings (Vestad et al., 2017). Although NTA camera level and detection threshold were determined as significant factors in the quantification of liposomes, changing these variable settings were less prominent for sEV quantification (Filipe et al., 2010; Gardiner et al., 2013; Maas et al., 2015). Sample heterogeneity, operator and software version were reported as other factors affecting the accuracy of NTA measurements (Vestad et al., 2017; Bachurski et al., 2019).

We will build on and expand these observations, specifically in relation to neat serum and pericardial fluid (PF) prepared from cardiovascular patients, with the intention of producing a “how to” guide for analysing these samples using NTA.

When analysing neat biofluids, particularly the ones deriving from blood, it must be taken into account that the size of sEVs and lipoproteins overlap partially. This is true for the following classes of lipoproteins: chylomicrons (75 nm–1.2 μm), chylomicron remnants (30–80 nm), very low density lipoproteins (VLDL: 30–80 nm) and intermediate-density lipoproteins (ILDL: 25–35 nm) (Feingold and Grunfeld, 2000).

One potential use for sEVs in the clinic is as biomarkers of disease, particularly through the extraction of sEVs from bodily fluid as a “liquid biopsy.” It is therefore important to standardise methodologies in place in order to demonstrate this. In many cases the biomarker studies involving sEVs have focussed on their molecular cargo, especially microRNAs (miRs) (Alegre et al., 2016; Manier et al., 2017; Selmaj et al., 2017). This aspect has considerable potential, but it might necessitate sEV extraction followed by molecular analyses, thus adding complexity and delay between taking the sample and obtaining the result. This is not ideal when an early diagnosis or prognostic indicator is necessary, such as in patients presenting with a suspected myocardial infarction, or to assess the risk of early complications following cardiac surgery. These and other situations are where, potentially, acute changes in sEV concentration or their size could be used (Cappello et al., 2017). We demonstrated, for example, that dramatic changes in plasmatic sEV-size particle concentrations could be detected in patients undergoing coronary artery bypass graft surgery (CABG) under cardiopulmonary bypass (CPB). We also demonstrated that, following surgery, the size of these particles changed and that they contained miRs (such as miR-1 and miR-133a/b) known to be enriched in the myocardium and whose concentration also increased significantly in both the plasma and plasma-derived sEVs (Bachurski et al., 2019). It is therefore conceivable that the plasma nanoparticle changes were caused by their induced release by the myocardium as a response to the ischaemia/reperfusion injury induced by the surgery. In line with this, both the sEV plasma concentration (measured by NTA) and the cardiac miRs in the sEVs were highly correlated with high sensitivity cardiac troponins (hs-cTNs), the gold standard biomarker for myocardial injury (Emanueli et al., 2016). We used neat plasma samples when carrying out NTA in this study, which is unusual among sEV studies, as others have extracted the sEVs from the plasma first (Vicencio et al., 2015). Our approach presents additional challenges when using NTA, such as the presence of a multitude of other particles and proteins in the sample that can confuse the tracking software. However, the currently available protocols for sEVs extraction are intrinsically associated with sEV loss (Lobb et al., 2015; Patel et al., 2019) and, especially in the case of plasma or serum, they have not be able to resolve the problem of protein and lipoprotein contaminations. Moreover, for potential applications in an acute bedside-testing type setting, the minimal handling offered by analyses on neat biofluid is remarkable.

There are a number of potential sources of error that should be taken in consideration when designing protocols for studies involving clinical samples using an NTA system. These have not been systematically demonstrated. For example, the handling of a sample from the immediate point at which it is taken has been suggested to introduce variability in the sEV content (Witwer et al., 2013). Similarly, it has also been suggested that storage and freeze-thaw cycles could have an impact on sEV integrity and aggregation (Bosch et al., 2016). This does not appear to be conclusive but, if true, has the potential to lead toward additional variability in results, particularly if samples are not handled consistently. We have thus carried out a thorough investigation such that we are able to suggest important methodological considerations for the quantification of nanoparticles in bodily fluids using some of the most up-to-date software and hardware available for NTA.

## Methods

### General Setup

All experiments were carried out using a NanoSight NS300 (Malvern PANalytical, Malvern, United Kingdom), with NTA software version 3.2 (Malvern Instruments). All samples were diluted using Gibco phosphate buffered saline (PBS, catalogue number 14190094) (Thermo Fisher, Paisley, United Kingdom) except where otherwise noted. The PBS was filtered prior to use using a Millex 33 mm, 0.22 μm syringe filter unit (Merck, Nottingham, United Kingdom). Samples were vortexed briefly, filtered using a 4 mm, 0.22 μm Millex syringe filter unit (Merck), except where otherwise noted, then taken up into a 1 mL BD Plastipak syringe (BD Biosciences, Wokingham, United Kingdom) and injected into the flow cell of the NanoSight NS300. Once loaded, the sample was passed through the system using a syringe pump at a rate of 50 arbitrary units (A.U.), decreased gradually through 500, 300, 200 and 100 A.U. Once stable, this flow rate allows the particles to pass across the field of view in approximately 8 s. A video would then be captured and processed (script attached, Supplementary File 1).

### Sample Collection

Samples were handled in accordance with the Declaration of Helsinki and the Human Tissue Act. The samples used in this study were collected as discarded tissue from different cardiac surgery patients operated on at our Centre and from healthy controls who provided informed consent. Samples were covered by ethical approvals from the UK National Research Ethic Service NRES (REC 10/H0107/63, 12/LO/1361, 13/LO/1687). Blood was collected in an SST Advance vacutainer (BD Biosciences) and transferred to the lab within 1 h, where it was double-centrifuged at 2240 × *g* and RT for 10 min to produce serum. The sera were quickly aliquoted and immediately frozen and kept at −80°C until use. Pericardial fluid was collected following the opening of the pericardial sac at the beginning of heart surgery. The PF was then moved to the lab and spun at 4°C at 300 × *g* for 5 min to and the supernatant collected. The supernatant was then spun again at 13000 × *g* for 5 min. The supernatant was collected and stored at −80°C until use.

### Investigation of Sample Composition

Nanoparticle counts were registered in parallel from the whole serum and sEVs isolated from the serum by size exclusion chromatography (SEC). sEVs were isolated as previously described (Beltrami et al., 2017) and tested for ApoA1 and ApoB by ELISA kits (ab108803, ab190806, Abcam, Cambridge, United Kingdom). This was performed in serum samples collected before and at 24 h following cardiac surgery (*n* = 6) to investigate if the serum nanoparticle counts followed the trend observed when measuring serum-derived sEVs. To completely explore the composition of the serum samples, we also considered the presence of high density lipoproteins (HDLs) and VLDL. To do that, we measured apolipoprotein A1 (ApoA1, which is paramount of the HDLs) and ApoB (characteristic from LDLs, IDLs, VLDLs and chylomicrons).

### Initial Preparation of the NTA

The optical glass of the laser module was initially wiped with a tissue dampened with:

1. 70% IMS.
2. MilliQ water.
3. 1% ethanoic acid (Sigma-Aldrich, Dorset, United Kingdom).
4. MilliQ water.

Following this, the low-volume flow cell was attached to the laser module and the system flushed with:

1. 1 mL 10% ethanol (Sigma-Aldrich).
2. 1 mL filtered Gibco PBS (Thermo Fisher).
3. 1 mL filtered Gibco PBS.

The machine was then considered ready for use.

### Standard Washing Procedure

The standard, non-automated, washing procedure adopted in this protocol was as follows:

1. 1 mL filtered Gibco PBS.
2. 1 mL filtered Gibco PBS.
3. 1 mL 1% ethanoic acid.
4. 1 mL filtered Gibco PBS.
5. 1 mL filtered Gibco PBS.

This was carried out prior to running any standard or sample.

### Standard Machine Settings

Unless otherwise stated, recordings were made using settings previously chosen to give the best contrast in our experience, using the NS300:

• Slider Shutter: 1300
• Slider Gain: 512
• Camera Histogram Upper Limit: 2470
• Camera Histogram Lower Limit: 130
• Syringe Pump Speed/AU: 50

Focus was set manually according to manufacturer’s instructions, ensuring that the maximum number of particles was in focus in the field of view, by maximising the central blob intensities and minimising the ring intensity (diffraction rings that can occur when particles are correctly focussed) to reduce non-zero-order peaks.

### Calibration and Reproducibility

We used a commercially available sEV standard consisting of sEVs derived from the plasma or serum of healthy human controls (HansaBioMed, Tallinn, Estonia) as a calibrator. The standard was diluted according to manufacturer’s instructions, aliquoted, then frozen in individual aliquots for single use. On each day of NTA use, a new aliquot was prepared into a working dilution suitable for running on the NanoSight by adding 5 μL of the standard to 995 μL PBS, giving a 1:200 dilution. Four 90-s videos were recorded under flow conditions and processed using Detection Threshold 9. This was repeated over 25 days to observe day-to-day variations, quantifying particle concentration and the number of particles in the 90–120 nm range.

### Serum Sample Preparation

Except for when testing diluents, serum was thawed for 30 min at RT and initially prepared for analysis by NTA at a 1:100 dilution by diluting 10 μL of sample with 990 μL PBS; 200 μL of this initial dilution was added to 800 μL PBS, to give a final 1:500 dilution. For the diluent experiment, 1:500 was not feasible for technical reasons (i.e., not sufficient to allow for particle detection when using water). Therefore, in this instance, serum samples were prepared at a 1:100 dilution using the diluent to be tested, then further diluted using 900 μL of the diluent and 100 μL of the sample to ultimately give a working dilution of 1:1000.

### Pericardial Fluid Sample Preparation

Pericardial fluid was thawed for 30 min at RT, then diluted 1:50. This was achieved by diluting 20 μL sample with 980 μL PBS. This dilution was also used for the diluent experiment.

### Investigating the Effect of Sample Freeze-Thaw Cycles on Particle Count

Each serum or pericardial sample was prepared and run according to our standard protocol. The original sample was then re-frozen at −70°C. The same sample was subsequently defrosted and run again using the same settings and again re-frozen. This was repeated twice more. Time-points were defined as: T_0_ – first defrosting of sample having been freshly frozen, T_1_ – second defrosting, T_2_ – third defrosting, T_3_ – fourth defrosting. Each sample thawed on the bench and was re-frozen overnight before thawing again the following day.

### Investigating the Effect of Different Diluents on Sample Particle Count

Initial manufacturer recommendations suggest that one should use water for diluting a sample for analysis with NTA, which might not be suitable for biological samples, as there is the possibility that biological vesicles could lyse due to osmotic potential. We investigated the effect on particle count of using different diluents to dilute both serum and PF samples. The diluents tested were the PBS we have used in our other experiments in this paper, simulated body fluid (SBF), Hanks Balanced Salt Solution (HBSS, Sigma-Aldrich, catalogue number H1387) and filtered, deionised water. SBF was prepared as previously described (Gu et al., 2010). HBSS was prepared according to the instructions supplied by the manufacturer. Four videos of 90s were acquired for each of the different diluents and processed using the standard settings above.

### Investigating the Effect of Sample Concentration

The sEV standard was initially prepared as above. Dilutions were made in filtered Gibco PBS (Table 1). The sample of the standard was then run and analysed on the NanoSight using the settings above, except that 3 90-s videos were recorded instead of 4 and the fold dilution recorded by the software as 1, rather than the actual fold dilution.

**TABLE 1:** Dilutions of plasma calibrator to determine linearity.

Fold dilution	1^st^ dilution		2^nd^ dilution (of 1^st^ dilution)	
	Vol standard (μL)	Vol PBS (μL)	Vol diluted standard (μL)	Vol PBS (μL)
20	50	950	–	–
40	25	975	–	–
50	20	980	–	–
66.67	15	985	–	–
100	10	990	–	–
125	8	992	–	–
142.875	7	993	–	–
200	5	995	–	–
250	4	996	–	–
333.33	3	997	–	–
400	10	990	250	750
500	10	990	200	800
800	10	990	125	875
1000	10	990	10	990
2000	10	990	50	950

### Investigating the Effect of Particle Masking

By “masking” we refer to a large particle hiding a smaller particle and thus excluding it from the total NTA count. Firstly, as a proof of principle for the issue of masking, 100 nm (NTA 4088) and 200 nm (NTA 4089) Latex Standard beads (Malvern) were diluted in PBS and used alone or in combination. 100 nm standards were diluted 1:1000, while 1:100 dilution was used for 200 nm standards. This meant that when 100 and 200 nm standards were combined, their ratio was 1:10 respectively. Three 30-s videos were recorded and all videos were processed using a Detection Threshold of 8. We hypothesise that a mixture of 100 nm and 200 nm particles would result in reduced counts in the sEV range due to masking.

The effect of particle masking was further explored using serum or PF samples, prepared as above. Four 90-s videos were acquired. The analysis was carried out using Detection Threshold 9. These were compared with the unfiltered equivalent sample, i.e. prepared in the same way, except that the filtration step was excluded. Again, we hypothesise that the unfiltered samples would have lower counts in the small EV range due to masking.

### Investigating the Effect of Video Length and Number of Videos

A serum or PF sample was prepared and four videos were recorded, using 30, 60, 90, 120, 150 and 180 s acquisitions. All videos were processed using a Detection Threshold of 9. A fresh sample was prepared for each test of video length. The flow cell was cleaned (as per the standard protocol) between each sample. The data recorded using videos of 90 s in length was also arbitrarily used to investigate the effect of the number of videos recorded on the results, comparing the standard deviation in particle concentration readings between acquisitions with 2, 3 and 4 recordings. We hypothesise that longer acquisition time and higher number of videos would result in smaller variability (standard deviation) of the sEV counts.

### Investigating the Effect of Changing Focus

A serum sample was prepared and four videos of 90 s each were recorded with the focus set correctly, as described above, then 10 and 30 A.U. below optimum. The videos were recorded consecutively, without pausing the syringe pump. Following the standard washing procedure, the experiment was repeated with a fresh sample, this time increasing the focus 10 and 30 A.U. above optimum. All videos were processed using a Detection Threshold of 9. The same experiment was carried out with PF, varying the focus by 10 or 30 A.U. in each direction.

### The Effect of Maximum Jump Distance

The “Maximum Jump” setting is used by the NTA software to determine how far it needs to “look” for a particle as it moves from one frame to the next in the video. The smaller the particle, the further it will move between frames and therefore a larger search area is required by the software to track it successfully. This is a setting which can be set to “Auto” and the software scans through the first 100 frames of the video to determine the optimum setting. However, there can be small changes between videos, so potentially this can introduce variability to the results. We investigated how manually keeping this setting constant affects the readings, compared to the automatic setting. Serum and PF samples were prepared as above and loaded into the machine. The same sample was then run four consecutive times on the same settings as above. The maximum jump setting was set to “Auto” for the first run, 14 A.U. for the second, 20 A.U. for the third and 10 A.U. for the fourth. Videos were analysed using Detection Threshold 9.

### Data Analysis

NanoSight Raw Data outputs were used in all analyses. All graphs show concentration/particle count data as mean ± SD. The magnitude of the effect of a variable is presented as a percentage change. Additionally, we present the variability in measurements, as observed by changes in the SD of the data. Whilst the focus is generally to observe the magnitude of changes induced by different settings, in some cases where a hypothesis was formulated in relation to a specific variable a statistical test was performed, using non-parametric tests (Mann–Whitney test, Wilcoxon test, Friedman test or Kruskal–Wallis test with Dunn’s *post hoc* test, as appropriate) with *p* < 0.05 indicating statistical significance.

## Results

### Samples

**FIGURE 1:**
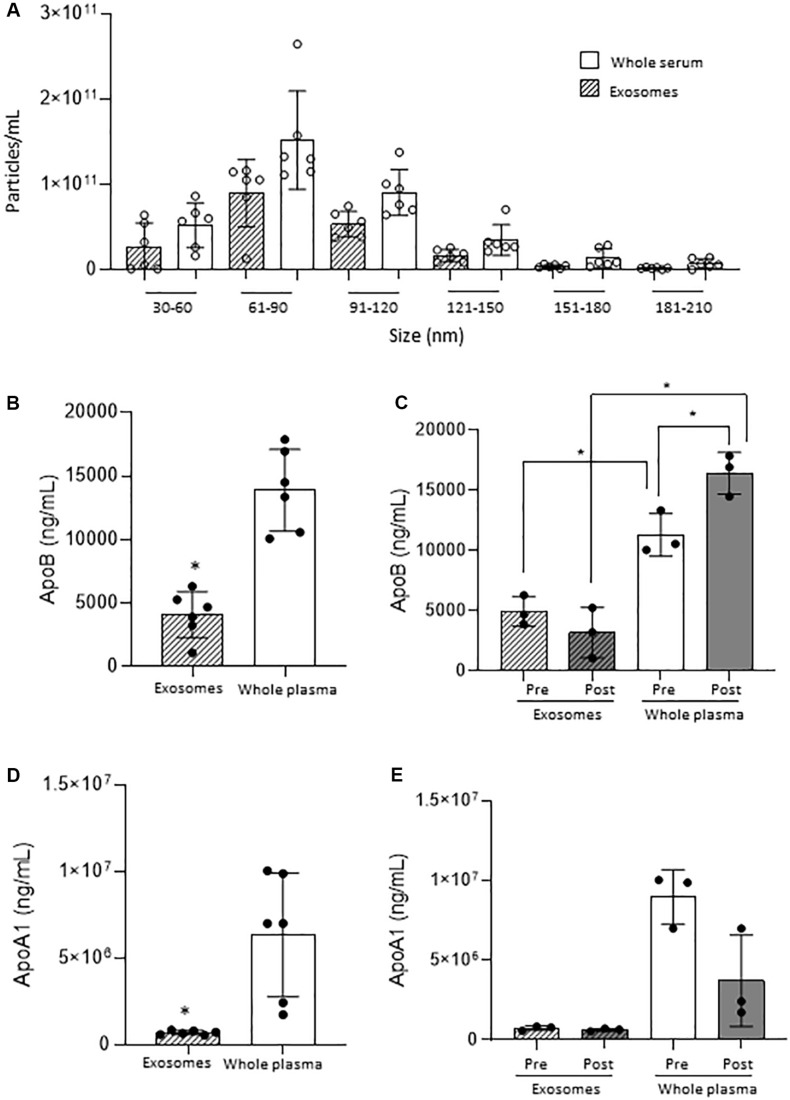
Small EVs vs. whole serum nanoparticles. **(A)** Comparison of nanoparticle concentration by size range. **(B)** ApoB quantification on small EVs and whole plasma in all samples. **(C)** ApoB quantification on small EVs and whole plasma in Pre- and Post-surgery samples. **(D)** ApoA1 quantification on small EVs and whole plasma in all samples. **(E)** ApoA1 quantification on small EVs and whole plasma in Pre- and Post-surgery samples. *p < 0.05.

Particle count results to characterise the serum samples revealed no significant difference when comparing whole serum measurements and isolated sEVs, suggesting that whole serum mimics the isolated sEVs. This was observed at all relevant particle ranges (30–60, 61–90, 91–120 and >121 nm) as shown in Figure 1. As confirmation of scarce lipoprotein presence on the isolated sEVs, ApoA1 and ApoB sharply decreased in isolated sEVs in comparison to the whole serum (Figures 1B,D). This was observed also when samples were divided according to timing of surgery (i.e., pre/post-surgery; Figures 1C,E), with ApoB interestingly showing increased values post-surgery.

### Calibration and Reproducibility

**FIGURE 2:**
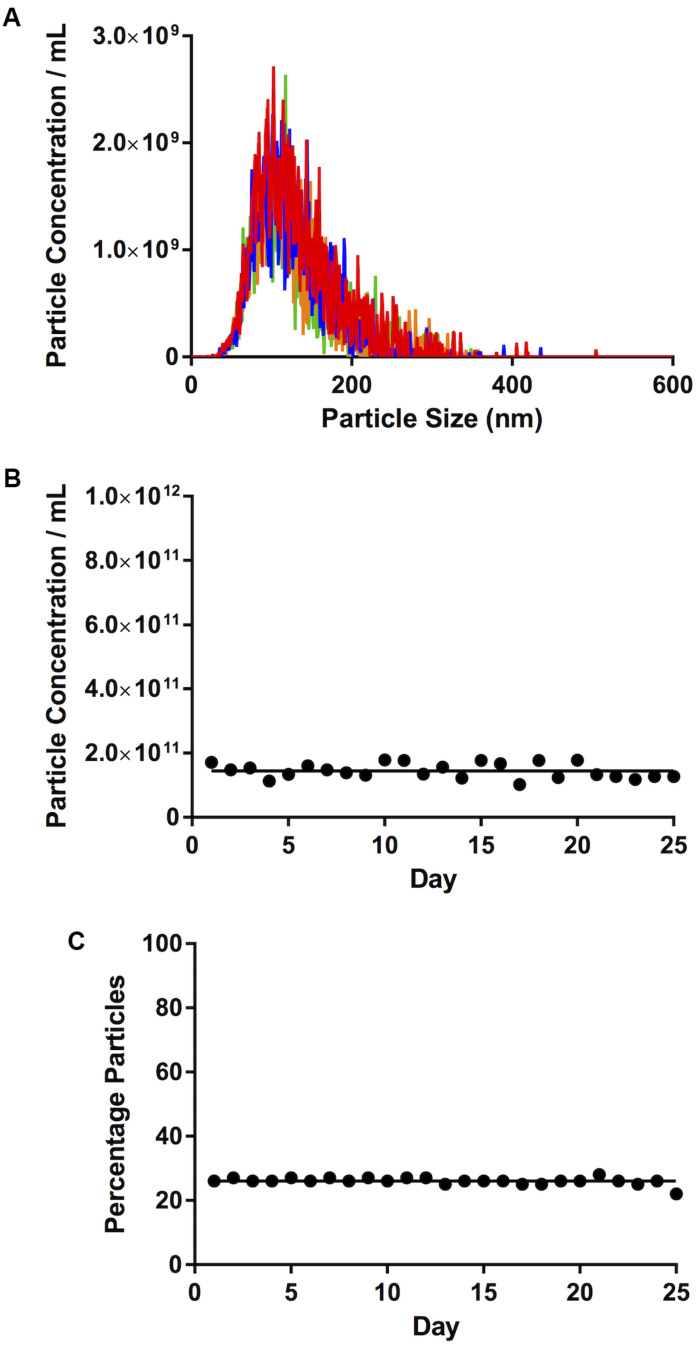
Calibration. **(A)** Traces of four consecutive videos, closely superimposed one on top of the other. **(B)** Total particle concentration of calibrator. **(C)** Percentage particles in the 90–120 nm range. Lines on **(B,C)** represent mean concentration and %, respectively.

Four runs using the sEV standard were carried out and the raw traces of each of the recordings were superimposed (Figure 2A). Importantly, there was little day-to-day variability in calibrator concentration over a 25-day time period (≤6%), in terms of either total particle concentration (Figure 2B) or percentage particles in the 90–120 nm range, as a reference size range (Figure 2C).

### Repeated Freeze-Thaw Cycles Tend to Increase in Particle Count

**FIGURE 3:**
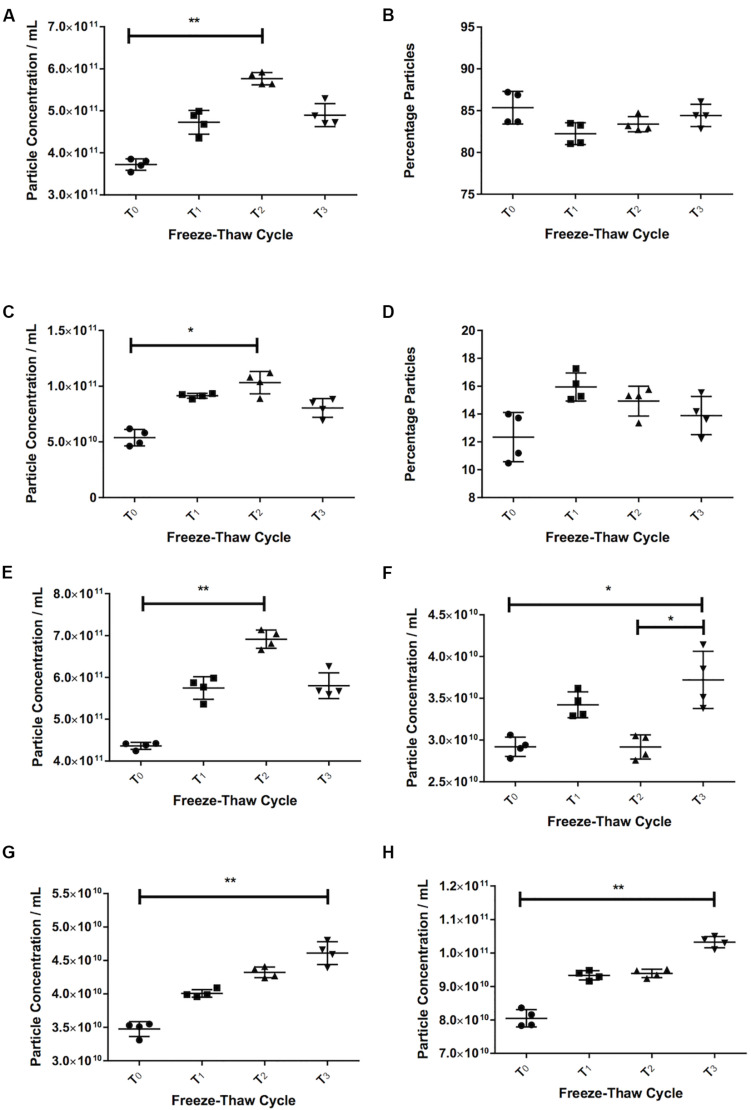
Freezing and thawing affects the recorded particle concentration. Serum particle concentration in the 30–120 nm **(A)** and 121–210 nm **(C)** range. Percentage particles in the 30–120 nm **(B)** and 121–210 nm range **(D)** in serum. Total particle concentration in serum **(E)**. Pericardial fluid (PF) particle concentration in the 30–120 nm **(F)** and 121–210 nm **(G)** and total particle concentration **(H)**. T_0_ = first defrosting of freshly frozen sample, T_1_ = second defrosting, T_2_ = third defrosting, T_3_ = fourth defrosting. *p < 0.05, **p < 0.01.

With serum, we saw an increase in the number of particles in the sEVs range (30–120 nm) with more freeze-thaw cycles [3.72 × 10^11^ ± 1.37 × 10^10^ particles/mL at T_0_, 4.73 × 10^11^ ± 2.83 × 10^10^ particles/mL at T_1_ (27% increase over T_0_), 5.77 × 10^11^ ± 1.46 × 10^10^ particles/mL at T_2_ (55% increase over T_0_, *p* = 0.006), Figure 3A]. The percentage of particles in this size range relative to the total particle concentration did not appear to vary (85% at T_0_, 82% at T_1_, 83% at T_2_, 84% at T_2_, Figure 3B). There was also an increase in the particle concentration in the 121–210 nm size range (*p* = 0.016 T_2_ vs. T_0_) (Figure 3C), and no differences in the percentage particles in this size range (Figure 3D). Therefore, there was an increase in the total number of particles, with a 58% increase between T_0_ (4.36 ± × 10^11^ 8.34 × 10^9^ particles/mL) and T_2_ (6.91 × 10^11^ ± 2.18 × 10^10^ particles/mL, Figure 3E, *p* = 0.006).

Pericardial fluid also showed a trend toward increasing particle counts with increasing freeze-thaw cycles. The 30–120 nm particles increased by 27% from 2.92 × 10^10^ ± 1.15 × 10^9^ particles/mL at T_0_ to 3.72 × 10^10^ ± 3.43 × 10^9^ particles/mL at T_3_ (*p* = 0.037, Figure 3F). Both the particle counts from 121 to 210 nm (3.48 × 10^10^ ± 1.11 × 10^9^ particles/mL at T_0_ to 4.61 × 10^10^ ± 1.71 × 10^9^ particles/mL at T_3_, *p* = 0.006, Figure 3G) and total particles (8.05 × 10^10^ ± 2.560 × 10^9^ particles/mL at T_0_ to 1.03 × 10^11^ ± 1.71 × 10^9^ particles/mL at T_3_, *p* = 0.006, Figure 3H) increased consistently (32 and 28% respectively).

### Different Diluents Give Different Results for the Same Sample

**FIGURE 4:**
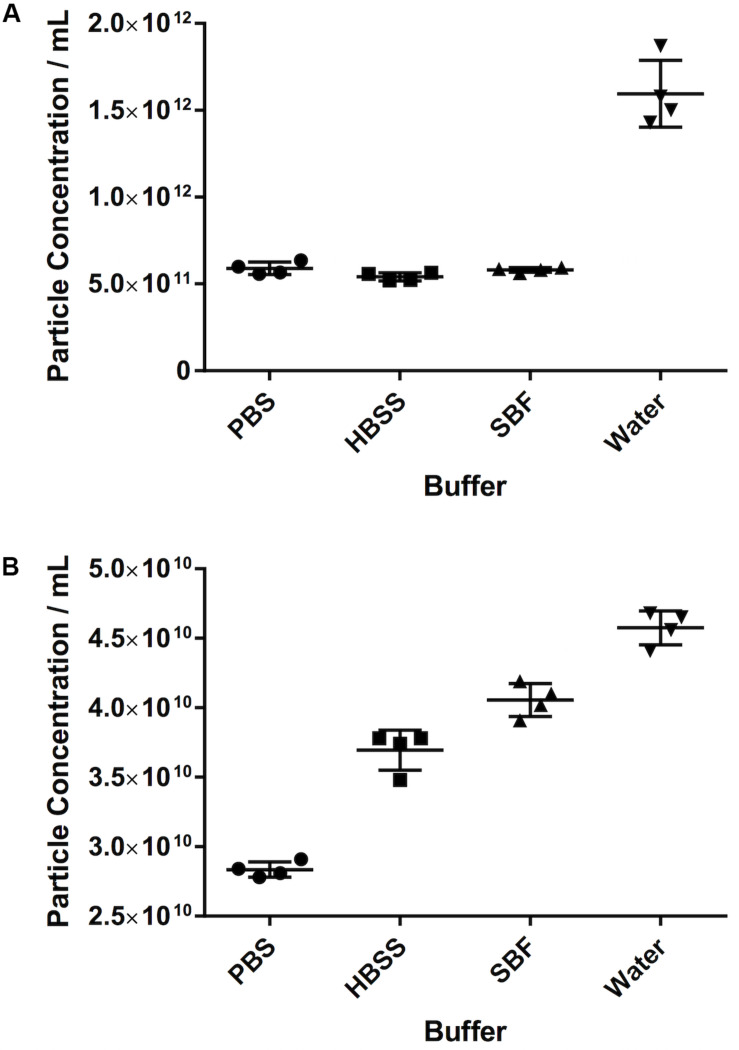
The effect of different diluents on particle count. **(A)** Serum, **(B)** pericardial fluid. Data shown is the particle concentration for the 30–120 nm size range with mean ± SD.

For both serum and PF, the particle count in water was substantially higher than in any of the other diluents. For serum in particular, the particle count was approximately threefold higher in water (15.9 × 10^11^ ± 1.9 × 10^11^ particles/mL vs. 5.8 × 10^11^ ± 1.3 × 10^10^ in SBF, 5.4 × 10^11^ ± 2.3 × 10^10^ in HBSS, and 5.9 × 10^11^ ± 3.6 × 10^10^ in PBS). For PF, particle count was 13% higher in water (4.6 × 10^10^ ± 1.2 × 10^9^ particles/mL) than SBF (4.1 × 10^10^ ± 1.19 × 10^9^ particles/mL), 24% higher than HBSS (3.7 × 10^10^ ± 1.45 × 10^9^ particles/mL) and 61% higher than PBS (2.84 × 10^10^ ± 5.57 × 10^8^ particles/mL). This is illustrated in Figure 4.

### Overcrowded Samples Result in Underestimation of Nanoparticles

**FIGURE 5:**
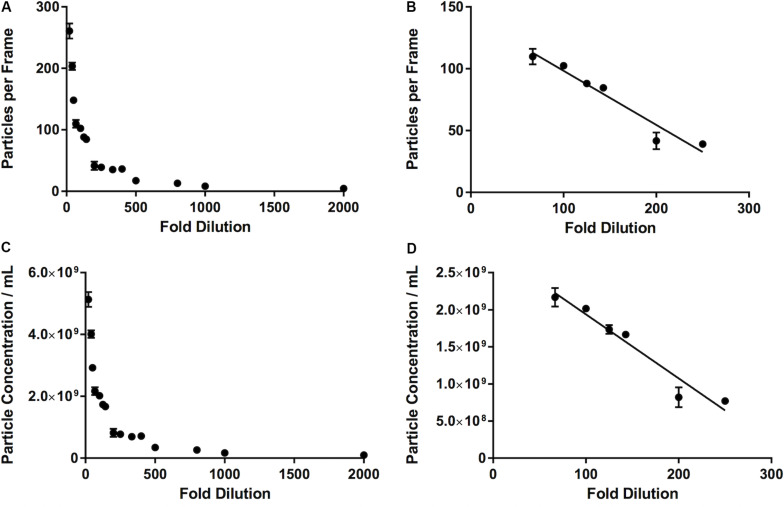
Relationship between number of particles per frame tracked by the software and a range of fold dilutions **(A)**, with a range of fold dilutions (66.67 to 250) where this relationship is linear (*R*^2^ = 0.93) and is largely within the range defined by the manufacturer (10–100 particles per frame) **(B)**. This pattern is repeated when fold dilution is compared with particle concentration **(C)**, where between a fold dilution of 66.67 and 250 this relationship becomes linear (*R*^2^ = 0.93) **(D)**.

We observed that, as the number of particles per frame increases, so does the particle concentration, in a linear fashion (*R*^2^ = 0.99). If we compare the fold dilution of the sample with either the particles per frame or measured particle concentration, this results in a non-linear curve (Table 2 and Figures 5A,C). This suggests that there comes a point where the machine over- or under-estimates the particle concentration, due to there being too few or too many particles per frame. There is a section of this curve which is, indeed, largely linear, between a fold dilution of 66.67 and 250 (*R*^2^ = 0.93, Figure 5D), corresponding to a particle-per-frame range 39–110 (Figure 5B).

**TABLE 2:** Number of completed tracks for each video of plasma calibrator linearity experiment.

Fold dilution	Video recording		
	1	2	3
20	73516	77511	75046
40	51540	50308	51963
50	73516	77511	75046
66.67	27977	25202	25327
100	26187	26263	26538
125	21032	19986	19967
142.875	19110	19241	19529
200	12516	10241	9769
250	8345	7930	7757
333.33	9593	9205	8742
400	8104	7725	6793
500	6772	5300	4202
800	6088	4239	3379
1000	6770	1975	1984
2000	1091	1295	*

*The concentration of this sample was so low that only two of the three videos gave any form of measurement.

### The Effect of Masking: Filtration Increases Particle Count

**FIGURE 6:**
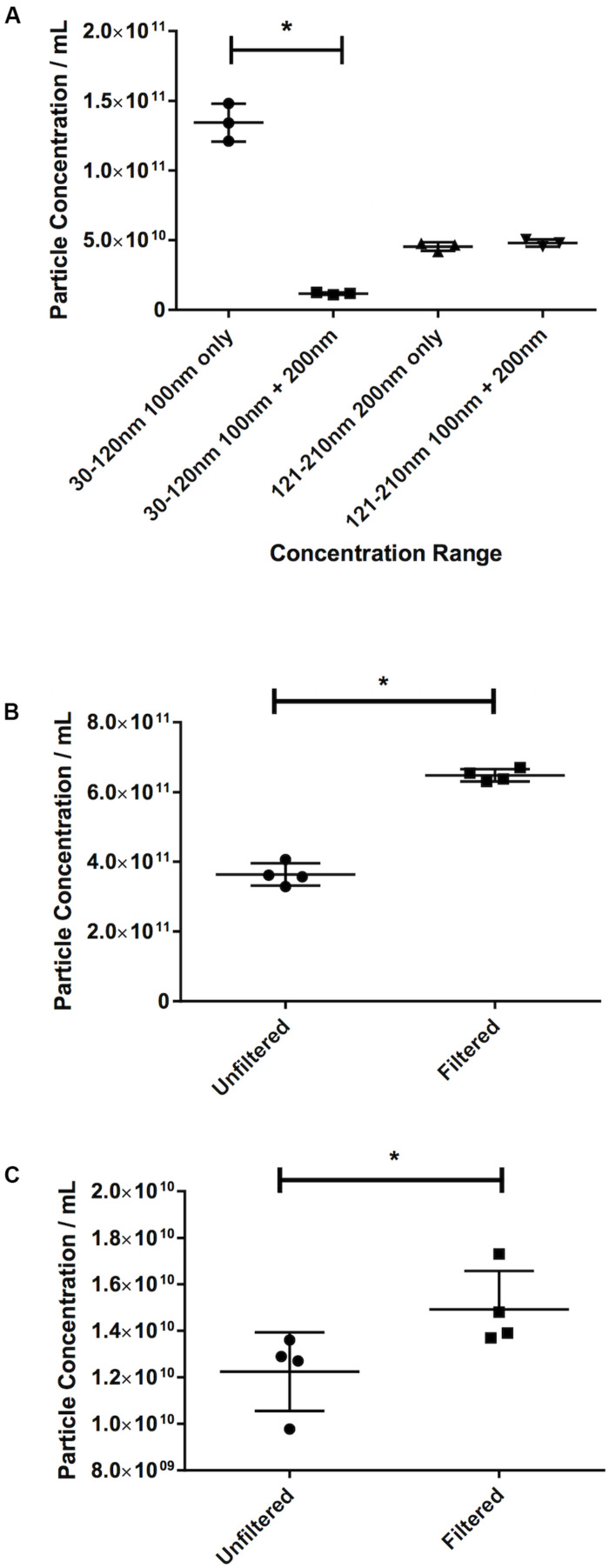
Filtration eliminates the masking effect of larger particles on smaller ones. **(A)** Concentration of latex beads in either monodisperse or polydisperse preparations. Concentration of particles in the 30–120 nm range in **(B)** serum or **(C)** pericardial fluid, in either unfiltered or filtered preparations. Data are shown as individual recordings and the mean ± SD. *p < 0.05.

The effect of masking was demonstrated in the first instance by the latex beads experiment. The recorded 30–120 nm particle concentration of latex beads decreases by 91% from 1.34 × 10^11^ ± 1.35 × 10^10^ particles/mL 100 nm beads alone, to 1.18 × 10^10^ ± 8.54 × 10^8^ particles/mL when mixed with 200 nm beads (*p* ≤ 0.013, Figure 6A). The 200 nm bead concentration, however, shows very little change (4.55 × 10^10^ ± 3.06 × 10^9^ particles/mL 200 nm beads alone vs. 4.80 × 10^10^ ± 2.50 × 10^9^ particles/mL when mixed with 100 nm beads, an increase of 6%).

This was confirmed by observing the effect of filtration. An increase of 78%, from 3.64 × 10^11^ ± 3.184 × 10^10^ particles/mL in the nanoparticle concentration in an unfiltered serum sample to 6.48 × 10^11^ ± 1.77 × 10^10^ particles/mL in a filtered sample (*p* = 0.029, Figure 6B) was recorded. Repeating the test on PF gave an increase in particle count in a filtered sample of 21% over an unfiltered sample (1.23 × 10^10^ ± 1.69 × 10^9^ particles/mL unfiltered vs. 1.49 × 10^10^ ± 1.65 × 10^9^ particles/mL filtered, *p* = 0.029, Figure 6C). This suggests that this effect is less noticeable in PF than in serum.

### Increasing Video Length, Up to a Point, Increases Particle Concentration

**FIGURE 7:**
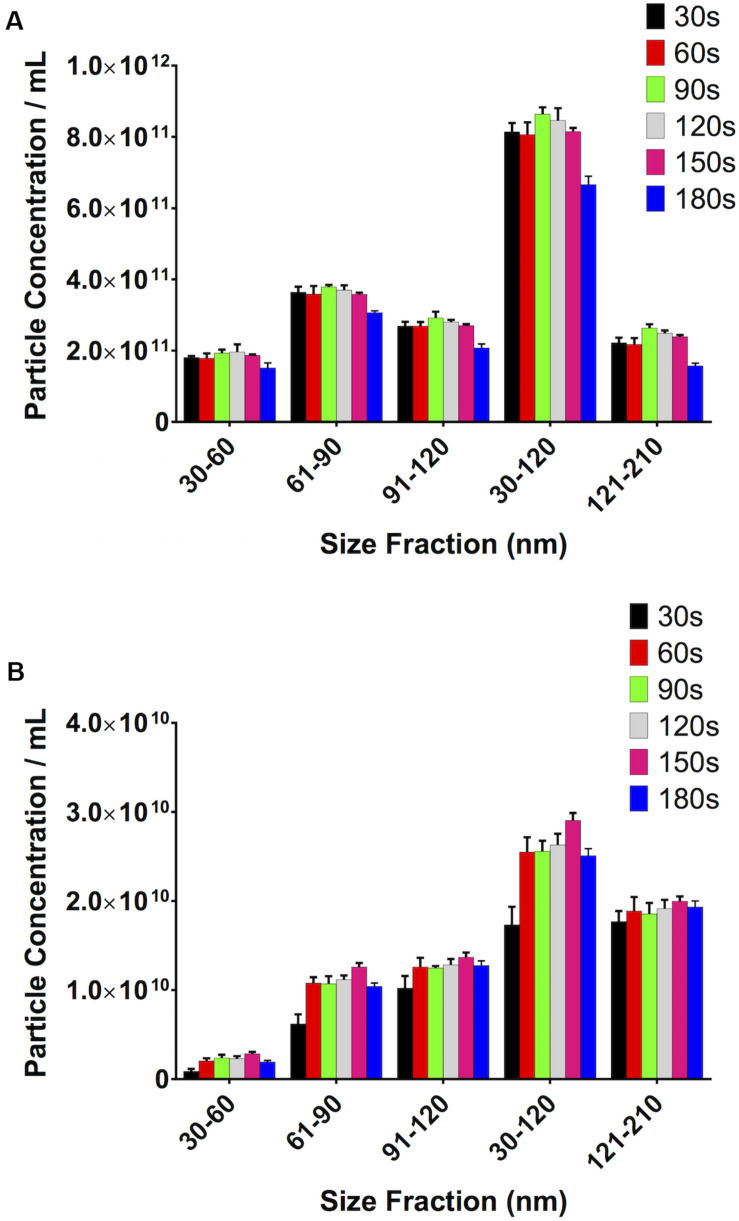
Video length has a noticeable impact on total particle concentration. **(A)** Serum, **(B)** pericardial fluid.

It stands to reason that the longer the video recordings are, the more events that will be captured and, therefore, the more opportunity the software has to track particles, thereby reducing the variability in the particle count between recordings and increasing the robustness of the result. Particle concentration in serum peaked at 90s video length (Figure 7A). Particle concentration in the 30–120 nm range varied by 6% from 30s to 90s. Interestingly, the number of counted particles begins to decrease beyond 90s, with a 30% decrease being observed for a 180s video, compared to 90s. The particle count at 90s also saw the lowest SD (1.93 × 10^10^). Repeating this experiment using PF gave similar results (Figure 7B). The highest particle count in the 30–120 nm range in this case was given by recordings of 150s, which were 12% higher than those calculated by 90s videos, however with a higher standard deviation (1.63 × 10^9^ vs. 1.17 × 10^9^ particles/mL).

### The Number of Recordings Has a Limited Impact on Particle Count

Overall, low variability between particle counts in the sEV range was observed when testing the effect of multiple recordings. We saw that with the PF, the SD remained very similar with an increasing number of videos. There was, however, an increase in the standard deviation with an increase in the number of videos when looking at serum (Table 3). However, the SD was <2.5% of the total particle concentration for serum and <5% for PF. In both cases, the total particle count increased with the number of recordings.

**TABLE 3:** Changes in particle concentration and standard deviation in pericardial fluid and serum samples with different numbers of videos recorded.

	Pericardial fluid		Serum	
Video number	Particle concentration	*SD*	Particle concentration	*SD*
2	2.54E + 10	9.90E + 08	8.48E + 11	2.83E + 09
3	2.59E + 10	1.16E + 09	8.56E + 11	1.40E + 10
4	2.56E + 10	1.19E + 09	8.64E + 11	1.93E + 10

### Small Changes in Focus Do Not Alter Particle Count

**FIGURE 8:**
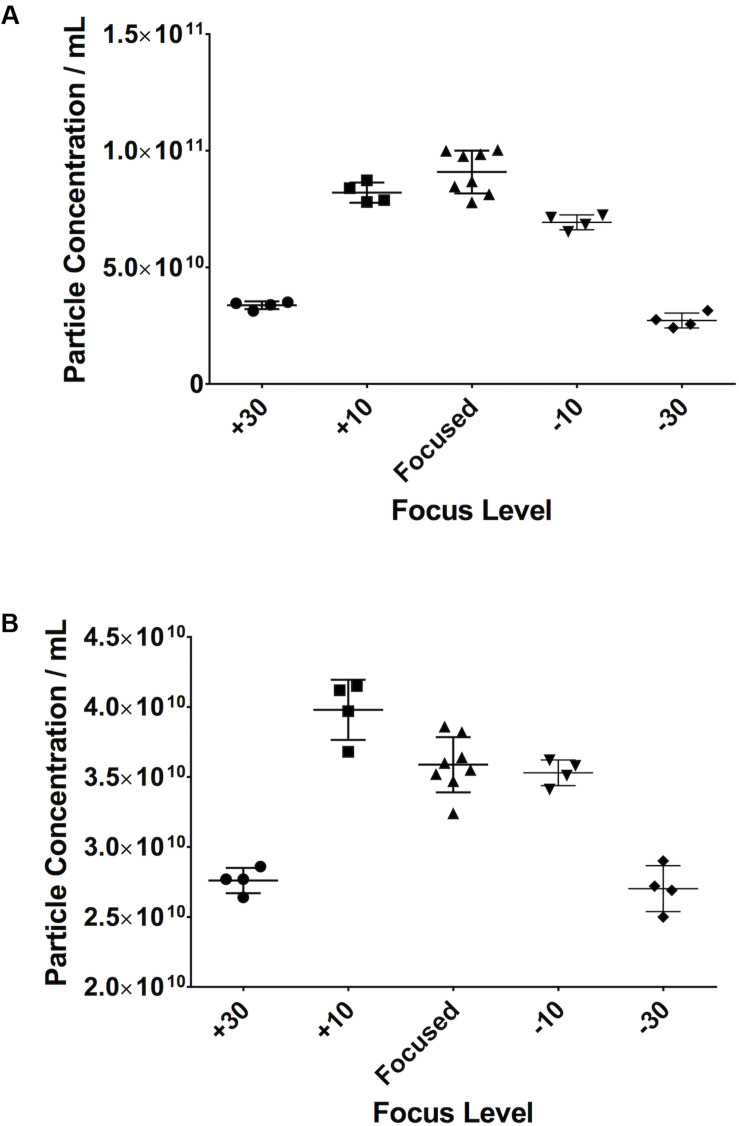
Small changes in focus levels do not have an impact on recorded particle concentration. Serum **(A)**, pericardial fluid **(B)**.

When looking at serum samples (Figure 8A), if the focus is increased a small amount away from optimum, i.e., 10 A.U. in our study, there is a 10% decrease in particle count. If it is decreased by the same value, there is a 22% decrease. However, if one moves more substantially away from focus (±30 A.U.), one sees a 63% drop in particle count on increasing the focus and a 70% decrease when decreasing it. The same is seen with PF (Figure 8B) where, if one substantially deviates from the optimum focus, there is a 22% drop in particle count when increasing the focus and a 25% drop on decreasing it. Examples of screenshots from under-, over-, and focussed videos (pericardial fluid) are shown in the Supplementary Figure 1.

### Small Variations in Maximum Jump Distance Do Not Affect Measurements

**FIGURE 9:**
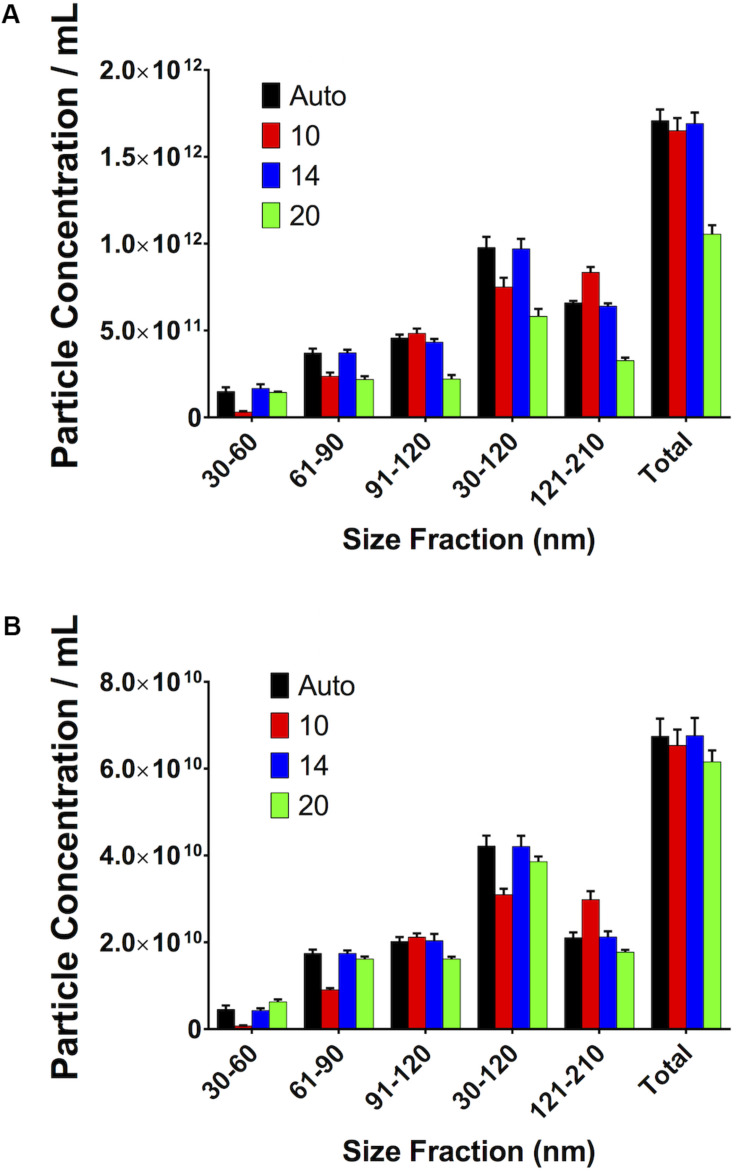
Small, reasonable changes in “Maximum Jump” settings do not affect concentration measurements. **(A)** Serum, **(B)** pericardial fluid.

If the Maximum Jump setting is manually altered to a setting close to that which the software would automatically select (i.e., 14) there is no difference in particle count. However, if the setting is moved further away from this optimal setting in either direction (i.e., 10 or 20), then variations in particle concentration are observed. This was seen in both serum (Figure 9A) and PF (Figure 9B).

## Discussion

Our study has investigated the way in which several different settings or preparation methods immediately prior to sample analysis have an effect on the particle concentration shown by NTA using a NanoSight NS300 apparatus, seeking to compare the effect of the settings or preparations tested on the same sample.

We have shown that it is possible to use commercially available sEV standards that, when aliquoted and stored in the same way, produce repeatable results when run on the NanoSight. If these are run over the duration of an experiment (days/weeks/months), they can be used as a calibrator. In our hands these sEVs appear stable over the course of several months when stored at −80°C.

We have also used these commercials EVs to investigate the linearity of the measurements made by the NanoSight. It is important, particularly in the case of neat human samples, to appreciate that there will be variability between patients. This has implications for the development of any technique which analyses neat samples, as it will have to be able to take into account this large variability. We have shown that there is a limit to with the NanoSight where the increase in particles per frame no longer gives a linear increase in the particle concentration. This is in line with the recommendations of the manufacturer and is likely due to a similar effect to that which we see with large particle masking. Here, with these particular samples, we suggest that this range is between approximately 40 and 110 particles per frame. In order to ensure that one is operating in this range, it may be necessary to change the dilution of the sample (e.g., due to variability between samples derived from different patients). Furthermore, it is possible that this varies from sample type to sample type. It is, therefore, perhaps prudent for the reader to carry out an experiment similar to that which we have carried out in this study to determine the best dilution for their samples. If on the one hand best practice indicates to apply the same protocol to all samples in a study, in the event of observing an overcrowded sample when running consecutive samples on the NTA the suggestion would be to adjust the concentration and repeat the measurement, to ensure that particle concentration is not under-estimated for that particular sample. This consideration applies particularly to studies involving patient-derived samples where variability between patients could be very high (e.g., between different conditions).

There have been suggestions that sEVs can be degraded or can aggregate when subjected to repeated freeze/thaw cycles (Zhou et al., 2006; Muller et al., 2014; Bosch et al., 2016). Our data suggests that the number of particles in the exosomal range will increase with repeated freezing and thawing. Potentially, this is due to the rupture of larger particles into smaller ones that are then counted in the sEV size range and/or to particles smaller than the sEV size range, which aggregate and are then counted within the larger size range. Therefore, it is important to bear this in mind when processing samples and the reader is encouraged to both minimise freeze-thaw cycles and to treat all samples in the same way.

Once the samples have been defrosted, they need to be diluted in the same manner, at the same concentration. Good experimental practice dictates that the same pipette type should be used for making up the same part of the dilution for all the samples in a given experiment.

The presence of larger particles has been reported to affect the quantification of smaller (<150 nm) extracellular vesicles in neat platelet-free plasma even using different techniques (e.g., Tunable Resistive Pulse Sensing) (Mørk et al., 2016) and indeed this is a concern for NTA. This issue can be mitigated by filtering samples with a 0.22 μm filter prior to running through the machine, although we accept that there may be some degradation of larger particles into smaller ones caused by this process. It is interesting also to note that the effect of filtering may depend on sample type. The biofluids we present here – serum and PF – are two contrasting examples. Serum, in our hands, is a particularly difficult sample to run on NTA and very much requires filtration prior to running. Pericardial fluid is less difficult, and one might avoid filtration in many cases. However, if one wishes to compare data between patients one needs to treat the same sample type in the same way across different patients. Therefore, we recommend filtering all samples as a matter of course.

The NanoSight must be focussed accurately before commencing any recordings, with some degree of tolerance. For example, we demonstrated that, with serum, an increase of 10 A.U. has only a 10% effect on particle count. However, if this is increased to 30 A.U., the particle count decreases by 63%. It is also interesting to note that the effect that this has varies depending on sample type.

Video length has an impact on the quality of the results obtained. We have shown that, up to a point, longer video acquisition allows for capturing more particles, whilst a shorter video would be more likely be affected (e.g., by an artefact due to an aggregate) and any small variation would have a bigger impact on the final total count. On the other hand, longer video acquisitions result in decreased particle counts. One can speculate that this may be due to some settling of the particles in the syringe over time, and/or their binding to the sides of the syringe. Peak values were recorded using different video lengths for the two different sample types used, but if one also looks at the standard deviations of the recorded values, 90 s videos gave the smallest SDs in both sample types. However, we suggest to the reader that it is perhaps prudent to run a test as we have done in this study to determine the optimum video length.

The number of videos recorded and small, reasonable variations in the Maximum Jump setting did not seem to have large effects on the results. One would again advocate consistency across measurements, and not altering the Maximum Jump from default settings.

With regards to camera gain settings, these were intentionally not tested as in our opinion they have been sufficiently discussed elsewhere (Filipe et al., 2010; Gardiner et al., 2013; Maas et al., 2015).

With regards to data presentation, we have shown particle concentrations as both absolute numbers of particles, as well as percentage particles relative to the total concentration. The total concentration is the total number of particles measured by the NanoSight in a given measurement. From the perspective of using this technology on patient-derived samples, this is useful if, for example, a given acute situation results in an increase in the number of sEVs released. The presentation of the results as a percentage of the total particles, on the other hand, can give a different and, possibly, complementary picture to that demonstrated by the absolute quantification. Presenting the results in this way potentially allows a change in the size of the particles released due to a given stimulus to be observed. Indeed, in our cardiac surgery study we saw both these effects (Emanueli et al., 2016).

We have included a sample script for the NTA software (Supplementary File 1), along with a sample Standard Operating Procedure (Supplementary File 2). It is important to note that while we have investigated the effect of sample handling inasmuch as how freeze-thaw cycles affect the results obtained through NTA, a complete discussion of sample handling is beyond the scope of this work and has, indeed, been investigated elsewhere (Witwer et al., 2013). However, it appears that when analysing samples using NTA, the adage “junk in, junk out” applies. It is, therefore, of the utmost importance that the reader appreciates that poor (or at least inconsistent) sample handling can have a considerable detrimental effect on NTA results.

There is documented evidence to suggest that lipoproteins do, partly, fall into the same size range as small EVs (Dragovic et al., 2011; Sódar et al., 2016; Mørk et al., 2017; Karimi et al., 2018) and would therefore interfere with the counts made by the NanoSight. Unfortunately, the tested apparatus does not have the capability to use fluorescence staining, so we are unable to use a fluorescent membrane dye to discern sEVs from lipoproteins. Data gathered as part of our sample characterisation revealed that whole serum mimics isolated sEVs; we did not observe a significant difference in particle concentrations between whole serum and isolated sEVs. Moreover, we detected low level of lipoproteins’ proteins (ApoA1 and ApoB) in serum-extracted sEVs. This is relevant to report because lipoproteins are potential contaminants of isolated sEV preparation (Grigor’eva et al., 2017). Whilst this study is still potentially confounded by the presence of lipoproteins, given that we have used neat biofluids, its goal of assessing different machine settings on human-derived samples is fulfilled and the observations on machine settings remain valid.

In conclusion, we have investigated the effect of several factors that could have an effect on the results produced by NTA using the NanoSight NS300 system, with a focus on clinically-derived samples. We suggest that the factors that must be controlled when running experiments on this system are:

1. Freeze thaw cycles of the sample being run,
2. Length of recorded videos,
3. Filtering samples prior to running on the machine to prevent masking of smaller particles by larger ones,
4. The number of particles per frame in the video recorded and whether this is in the range of particles per frame where the concentration is calculated linearly by the NanoSight,
5. Using a saline-based diluent (e.g., PBS) to dilute the sample before running.

The aspects that are less important, but that we advise the reader to be aware of are:

1. Focus – small variations are acceptable and will not have a major impact on results, however this can vary depending on the sample type.
2. “Maximum Jump” settings can set to default values.
3. The number of videos has a minimal impact on particle counts, however, we would encourage the reader to record multiple videos (minimum 3), as this will increase confidence in their results and allow a more representative volume of the total sample to be analysed.

The use of a calibrator is advised. In this case we have shown the suitability of commercially available small EVs for this purpose. If these are prepared, aliquoted and frozen on the same day they can be used throughout the experiment as a reference to demonstrate that the machine is producing repeatable results. This allows the reader to be confident that their data from 1 day to the next can be compared.

## Data Availability Statement

The datasets generated for this study are available on request to the corresponding author.

## Ethics Statement

The studies involving human participants were reviewed and approved by UK National Research Ethic Service NRES (REC 10/H0107/63, 12/LO/1361, 13/LO/1687). The patients/participants provided their written informed consent to participate in this study.

## Author Contributions

AIS carried out the measurements. AIS, GB, and CE planned the experiments. SA, PC-M, AS, AC, CB, and PP provided expert advice in interpreting the results. AIS and GB drafted the manuscript. All authors contributed significantly to critically revising the manuscript.

## Conflict of Interest

PC-M and AS were employed by company Malvern Instruments, Ltd. The remaining authors declare that the research was conducted in the absence of any commercial or financial relationships that could be construed as a potential conflict of interest.

## Supplementary Material

FIGURE 1 Screenshots of under-focussed, focused, and over-focussed videos taken when analysing pericardial fluid samples.

FILE 1 An example script for NTA 3.2 software.

FILE 2 An example Standard Operating Procedure for running clinical samples using NTA.
