# Supplementary figures and images for: Analysis of Neat Biofluids Obtained During Cardiac Surgery Using Nanoparticle Tracking Analysis: Methodological Considerations
Source: Front Cell Dev Biol. 2020 May 25;8:367. doi: 10.3389/fcell.2020.00367 (PMC7262431; doi:10.3389/fcell.2020.00367)

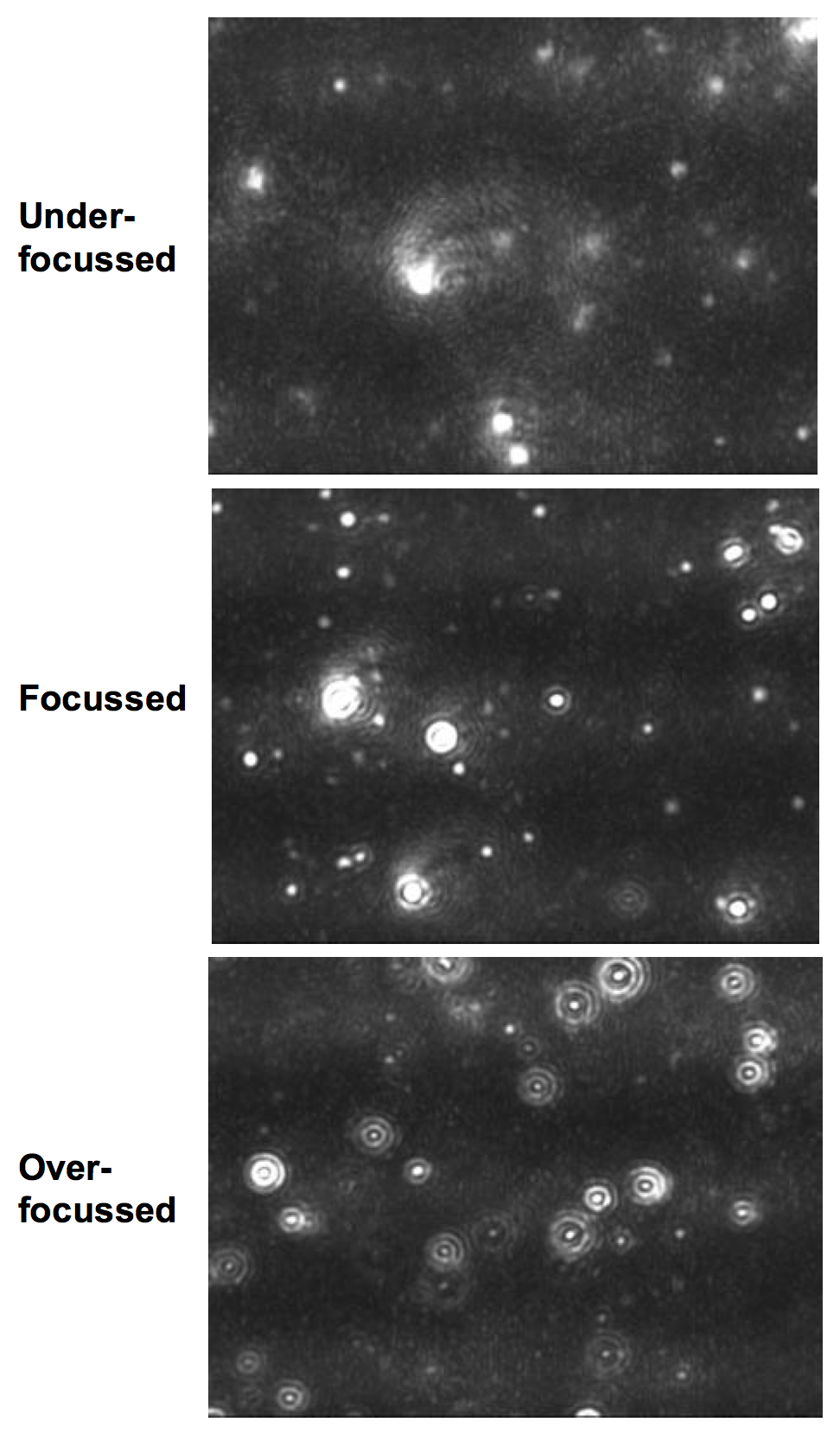

Supplement: FIGURE 1 — Screenshots of under-focussed, focused, and over-focussed videos taken when analysing pericardial fluid samples. [file Image_1.TIFF]
