# Supplementary material for: Analysis of Neat Biofluids Obtained During Cardiac Surgery Using Nanoparticle Tracking Analysis: Methodological Considerations
Source: Front Cell Dev Biol. 2020 May 25;8:367. doi: 10.3389/fcell.2020.00367 (PMC7262431; doi:10.3389/fcell.2020.00367)
Supplement: FILE 1 — An example script for NTA 3.2 software. [file Data_Sheet_1.PDF]

Supplementary File 1: Example Script

RECORDDILUTION 1000

SETVISCOSITY WATER

SETTEMP 25

SYRINGELOAD 50

CAMERASHUTTER 1300

CAMERAGAIN 512

CAMERALOLIM 130

CAMERAHILIM 2470

DELAY 1

REPEATSTART

CAPTURE 90

DELAY 1

REPEAT 3

SYRINGESTOP

TEMPERATURECONTROLOFF

DETECTTHRESHOLD 9

PROCESSBASIC

EXPORTRESULTS
