# Supplementary material for: Analysis of Neat Biofluids Obtained During Cardiac Surgery Using Nanoparticle Tracking Analysis: Methodological Considerations
Source: Front Cell Dev Biol. 2020 May 25;8:367. doi: 10.3389/fcell.2020.00367 (PMC7262431; doi:10.3389/fcell.2020.00367)
Supplement: FILE 2 — An example Standard Operating Procedure for running clinical samples using NTA. [file Data_Sheet_2.PDF]

Supplementary File 2: Standard Operating Procedure

1. Clean optical glass with 70% EtOH, water, 1% acetic acid and water before assembly. Dry with air duster.

2. Clean NTA fluidics with 1mL 10% EtOH and 2mL PBS once assembled.

3. Defrost sample at RT.

4. Meanwhile, carry out standard washing protocol: 2mL filtered PBS, 1mL acetic acid, 2mL filtered PBS.

5. Prepare required dilution of sample.

6. Vortex and filter using a 4mm 0.22µm filter.

7. Inject sample into flow cell to 0.55mL marker on syringe.

8. Adjust focus and run sample with example script using:

   Slider Shutter: 1300
   Slider Gain: 512
   Camera Histogram Upper Limit: 2470
   Camera Histogram Lower Limit: 130
   Syringe Pump Speed/AU: 50
   Video Capture Duration: 90s

9. During analysis, maintain Detection Threshold 9.

10. Repeat from step 3.

11. At the end of the day carry out standard cleaning protocol, then 1mL 10% EtOH and 2mL water.

12. Detach top plate from laser module and clean with 70% EtOH, water, 1% acetic acid and water. Dry with air duster.
